# Fine Mapping of Genetic Variants in *BIN1*, *CLU*, *CR1* and *PICALM* for Association with Cerebrospinal Fluid Biomarkers for Alzheimer's Disease

**DOI:** 10.1371/journal.pone.0015918

**Published:** 2011-02-09

**Authors:** John S. K. Kauwe, Carlos Cruchaga, Celeste M. Karch, Brooke Sadler, Mo Lee, Kevin Mayo, Wayne Latu, Manti Su'a, Anne M. Fagan, David M. Holtzman, John C. Morris, Alzheimer's Disease Neuroimaging Initiative, Alison M. Goate

**Affiliations:** 1 Department of Biology, Brigham Young University, Provo, Utah, United States of America; 2 Department of Psychiatry, Washington University School of Medicine, St. Louis, Missouri, United States of America; 3 Department of Neurology, Washington University School of Medicine, St. Louis, Missouri, United States of America; 4 Hope Center for Neurological Disorders, Washington University School of Medicine, St. Louis, Missouri, United States of America; 5 Charles F. and Joanne Knight Alzheimer's Disease Research Center, Washington University School of Medicine, St. Louis, Missouri, United States of America; Mental Health Research Institute of Victoria, Australia

## Abstract

Recent genome-wide association studies of Alzheimer's disease (AD) have identified variants in *BIN1*, *CLU*, *CR1* and *PICALM* that show replicable association with risk for disease. We have thoroughly sampled common variation in these genes, genotyping 355 variants in over 600 individuals for whom measurements of two AD biomarkers, cerebrospinal fluid (CSF) 42 amino acid amyloid beta fragments (Aβ_42_) and tau phosphorylated at threonine 181 (ptau_181_), have been obtained. Association analyses were performed to determine whether variants in *BIN1, CLU, CR1* or *PICALM* are associated with changes in the CSF levels of these biomarkers. Despite adequate power to detect effects as small as a 1.05 fold difference, we have failed to detect evidence for association between SNPs in these genes and CSF Aβ_42_ or ptau_181_ levels in our sample. Our results suggest that these variants do not affect risk via a mechanism that results in a strong additive effect on CSF levels of Aβ_42_ or ptau_181_.

## Introduction

Alzheimer's disease (AD) is the most common form of dementia and is neuropathologically characterized by extracellular senile plaques containing amyloid beta (Aβ) and intracellular neurofibrillary tangles containing hyperphosphorylated tau protein. Mendelian forms of the disease are caused by mutations in the amyloid precursor protein (*APP*) gene and the presenilin 1 and 2 genes (*PSEN1* and *PSEN2* respectively). While only apolipoprotein E (*APOE*) has been clearly identified as a susceptibility gene in the more common form of AD, data from recent genome-wide association studies has implicated several other common risk variants [1], [2], [3], [4], [5], [6], [7], [8]. Variants in bridging integrator 1 (*BIN1*), clusterin (*CLU;* also referred to as *APOJ*), complement component receptor 1 (*CR1*) and phosphatidylinositol binding clathrin assembly protein (*PICALM*) have already been reported to show replicable association with risk for AD [5], [6], [7], [8].

Identifying associated variants is an important first step toward understanding novel aspects of the etiology of disease. Characterization of the mechanisms by which these variants, or other functional variants in strong linkage disequilibrium, influence risk for disease will provide a better understanding of the biology of disease. Initial publications for these novel, AD associated variants provided some hypotheses for each of the reported genes. Previously reported work suggests that CLU and APOE may have additive effects on Aβ deposition [9]. CR1 may contribute to Aβ clearance [10]. Convincing evidence for an Aβ-related mechanism for risk exists for both of these genes. Less is known about the effects of BIN1 and PICALM on Aβ or tau metabolism: BIN1 function may affect risk for AD by altering neuronal membranes and clathrin mediated synaptic vessel formation [8], [11] and changes in PICALM function result in perturbation at the synapse, possibly altering synaptic vesicle cycling and leading to altered risk for AD [12], [13].

In our previous work we have shown the utility of using two well-established cerebrospinal fluid (CSF) biomarkers for AD, 42 amino acid fragments of amyloid beta (Aβ_42_; decreased in AD) and tau phosphorylated at threonine 181 (a proxy for hyperphosphorylated tau; ptau_181_; increased in AD), as endophenotypes for genetic studies of AD [14], [15], [16], [17]. In this approach we test variants for genetic association with CSF levels of Aβ_42_ and/or ptau_181_ levels. In cases where risk variants have already been identified this approach allows us to validate or generate hypotheses regarding the biological mechanism of risk. We can also take advantage of the increased statistical power and decreased heterogeneity of the biomarker phenotype relative to qualitative clinical diagnosis to identify novel variants that affect biomarker levels and aspects of disease [18]. In our previous studies using this approach we have successfully validated hypothesized effects of rs2986019 in *CALHM1* on CSF Aβ_42_ levels [19], generated testable biological hypotheses for AD implicated variants [16], and identified novel variants in *MAPT* and *PPP3R1* that are associated with both biomarker levels and rate of progression of AD [14], [17]. In this study we use an endophenotype-based approach to test predictions of biological effects on Aβ_42_ levels for variants in *CLU* and *CR1* and to attempt to generate biological hypotheses of risk mechanism for *BIN1, CLU, CR1* and *PICALM*.

## Methods

### Samples

CSF for the Washington University in St. Louis (WU) series was collected from 407 individuals after overnight fasting. CSF collection and processing as well as CSF biomarker measurements were performed as described previously [20]. Characteristics of the sample, including a breakdown of demographic information in demented and non-demented individuals can be found in table 1.

**Table 1:** Sample characteristics.

		WU			ADNI	
	All	Cases	Controls	All	Cases	Controls
N	407	102	305	257	154	103
age (SD)	69 (10)	74 (8)	67 (11)	76 (7)	75 (8)	77 (5)
CDR	0 = 71% 0.5 = 17% 1 = 5.6% 2 = 0.4%	All>0	All = 0	0 = 40% 0.5 = 27% 1 = 28% 2 = 3%	All>0	All = 0
% female	62	46	67	56	59	50
%*ε4*pos	37	54	34	47	64	23
Aβ_42_ (SD)	1575 (244)	1429 (195)	1621 (240)	2173 (58)	2149 (46)	2208 (55)
ptau_181_ (SD)	162 (34)	183 (42)	156 (27)	234 (19)	240 (19)	224 (14)

Sample size (N), mean and standard deviation for age in years, Clinical Dementia Ratings (CDR), the percentage of females in the sample (%female), percentage of the sample that carries at least one *APOE ε4* allele (%*ε4*pos) and the mean and standard deviation for Aβ_42_ in pg/ml and ptau_181_ in pg/ml for the complete Washington University CSF sample (WU: All), cases and controls and the complete Alzheimer's Disease Neuroimaging Initiative (ADNI: All), cases and controls are shown.

1 analyzed with Innotest ELISA (Innogenetics, Ghent, Belgium).

2 analyzed with AlzBia3 (xMAP) assay (Innogenetics, Ghent, Belgium).

Data from 257 samples with biomarker data and either AD or cognitively normal diagnoses from the Alzheimer's Disease Neuroimaging Initiative (ADNI) were also used. Data used in the preparation of this article were obtained from the ADNI database (http://adni.loni.ucla.edu). The Principal Investigator of this initiative is Michael W. Weiner, M.D., VA Medical Center and University of California – San Francisco. ADNI is the result of efforts of many co-investigators from a broad range of academic institutions and private corporations, and subjects have been recruited from over 50 sites across the U.S. and Canada. The initial goal of ADNI was to recruit 800 adults, ages 55 to 90, to participate in the research — approximately 200 cognitively normal older individuals to be followed for 3 years, 400 people with mild cognitive impairment (MCI) to be followed for 3 years, and 200 people with early AD to be followed for 2 years.” For up-to-date information see www.adni-info.org. Sample characteristics, including age, clinical dementia rating, gender, *APOE ε4* status and mean and standard deviation of the CSF biomarkers can be found in table 1. ADNI phenotype and GWAS data are publically available (http://adni.loni.ucla.edu/). The genotypes from this study will be provided upon request to the authors.

Biomarker values in both samples were measured using internal standards and controls that ensure consistent and reliable measurements. Differences between the measured values in the WU and ADNI samples are likely to be due to differences in the antibodies and measurement technologies used for each series (e.g. standard ELISA with Innotest in the WU samples, Luminex with AlzBia3 in the ADNI samples). It is also possible that the inclusion of more AD cases and older individuals in the ADNI data or differences in the number of freeze thaw cycles prior to analysis (1 cycle for WU samples and 2 cycles for ADNI samples) accounts for some of the variation in the biomarker measurements. CSF biomarkers in the two samples show association with similar covariates [17], [19].

### SNP selection and genotyping

For each gene we downloaded the list of SNPs in the gene region (and approximately 500 kb of flanking sequence) from HapMap. These SNPs were then evaluated for putative functional effects using SNPseek and Pupasuite. SNPs with putative function and SNPs that showed association in the original published reports were designated as forced tags in the tagging algorithm in Haploview when an r^2^ cutoff of 0.8 was applied. A total of 283 SNPs were selected (see table S1 for a list of all SNPs in the study).

Genotyping was performed using Applied Biosystems OpenArray technology (http://www3.appliedbiosystems.com/cms/groups/mcb_support/documents/generaldocuments/cms_058198.pdf), a means of running multiple TaqMan assays together on one chip. 125 ng per sample was added to the reaction mix and spread over 64 assays, the chips were thermocycled as described in the linked protocol and imaged. Results were analyzed using the Applied Biosystems Genotyper software (https://products.appliedbiosystems.com/ab/en/US/adirect/ab?cmd=catNavigate2&catID=607267&tab=Literature). Samples were analyzed on a plate-by-plate basis in the context of all the samples to eliminate variation in calls between plates. SNPs that deviated from HW equilibrium (p-value threshold 0.001), had a genotyping rate lower than 95% or a minor allele frequency of less than 5% were removed. Samples with a genotyping rate lower than 95% were also removed. After application of these quality control criteria there were 664 samples and 233 SNPs.

### Analysis

Ptau_181_ levels were normally distributed after log-log transformation. Using stepwise regression analysis we identified age, *APOE* ε4 genotype and Clinical Dementia Rating (CDR) as significant covariates to be included in the model. Gender was not significantly associated with CSF ptau_181_, and was not included in the model. Association with genotype was tested using ANCOVA after adjustment for these covariates. For the combined analysis we also included site as a covariate. All analyses were also performed without CDR as a covariate but there were no qualitative differences in the results.

Aβ_42_ levels were not normally distributed even after a variety of transformations were applied. For this reason Aβ_42_ analyses were performed using permutation based testing in PLINK (1 million permutations) [21]. For the WU data the statistical analyses included age, CDR and *APOE ε4* genotype as covariates; the ADNI model included CDR and *APOE ε4* genotype. Age was not significantly associated with biomarker levels in the ADNI sample (due to lack of variation in age in the ADNI sample) and was therefore not included as a covariate for analyses of that sample alone. Site was included in the combined analysis in addition to age, CDR and *APOE* ε4 genotype. There were no qualitative differences in the results when run without CDR as a covariate.

The alpha level for this study using Bonferroni correction for 233 tests is 0.00021. A less conservative correction using the Eigen values of the SNP correlation matrix to estimate the effective number of tests yielded an adjusted alpha of 0.00022 [22], [23]. Using either adjusted alpha yields the same qualitative conclusions from these data.

Haplotype and set-based analyses were performed using PLINK with default settings [21]. The SNPs selected for fine mapping around each GWAS hit were defined as a set and 10,000 permutations were run using the same models as described previously for each phenotype.

### Power

Power for the overall F test in a one-way, three-group analysis of variance was calculated using proc power in SAS. The effect size, measured in “fold-difference” between the means at which power was estimated at 0.80 was calculated for minor allele frequencies from 0.10 to 0.50 and alpha levels of 0.05 and 0.00021 (the Bonferroni correction for 233 tests) assuming markers do not deviate from Hardy-Weinberg Equilibrium (table 2).

**Table 2:** Power analyses.

Minor allele frequency	Effect size when power = 0.80	
	alpha = 0.05	alpha = 0.00021
0.1	1.03	1.05
0.15	1.026	1.042
0.2	1.024	1.038
0.25	1.022	1.035
0.3	1.02	1.033
0.35	1.019	1.032
0.4	1.019	1.03
0.45	1.019	1.03
0.5	1.019	1.03

Power to detect genetic association. Power for the overall F test in a one-way, three group analysis of variance. The effect size, measured in “fold-difference” between the means at which power was estimated at 0.80 was calculated for minor allele frequencies from 0.10 to 0.50 and alpha levels of 0.05 and 0.00021.

## Results

We failed to detect significant association between the CSF biomarker levels and SNPs in *BIN1*. rs3820757 (p = 0.31), rs744373 (p = 0.44) and rs2276582 (p = 0.48) had the smallest p-value for association with CSF Aβ_42_ levels but did not show statistically significant association in the combined sample (WU+ADNI CSF samples, table 3). The three top hits in *BIN1* for CSF ptau_181_ did not show statistically significant association (table 4). The SNP identified in previous GWAS, rs744373, did not show an association with CSF ptau_181_ levels in the combined sample (p = 0.79; table 4). Set-based analyses of the 14 *BIN1* fine mapping SNPs were not significant for either biomarker phenotype (Aβ_42_ p = 0.42; ptau_181_ p = 0.37). Haplotype analyses also failed to identify significant association with Aβ_42_ and ptau_181_.

**Table 3:** Top hits and GWAS SNPs for CSF Aβ_42_.

SNP	Gene	WU	ADNI	Combined
rs3820757	*BIN1*	0.14	0.43	0.31
rs744373*	*BIN1*	0.45	0.32	0.44
rs2276582	*BIN1*	0.27	0.43	0.48
rs10216623	*CLU*	0.0011	0.81	0.011
rs2640734	*CLU*	0.05	0.07	0.036
rs17057419	*CLU*	0.09	0.38	0.056
*rs11136000* *	*CLU*	0.92	0.14	0.79
rs1048971	*CR1*	0.47	0.80	0.25
rs17258996	*CR1*	0.38	0.96	0.32
rs2296160	*CR1*	0.32	0.75	0.33
*rs6656401* *	*CR1*	0.55	0.72	0.63
rs7113656	*PICALM*	0.053	0.69	0.0090
rs11234454	*PICALM*	0.0088	0.34	0.01
rs10792828	*PICALM*	0.0074	0.021	0.011
*rs3851179* *	*PICALM*	0.64	0.52	1.0

Association with CSF Aβ_42_ levels. P-values for association between the top three hits and CSF Aβ_42_ levels in the Washington University (WU), Alzheimer's Disease Neuroimaging Initiative (ADNI) and Combined series.

*SNPs that are significant in previously reported genome-wide association studies are also shown, even when not ranked in the top three hits.

**Table 4:** Top hits and GWAS SNPs for ptau_181_.

SNP	Gene	WU	ADNI	Combined
rs9653202	*BIN1*	0.019	0.82	0.077
rs1060743	*BIN1*	0.46	0.075	0.093
rs6431221	*BIN1*	0.059	0.74	0.10
*rs744373* *	*BIN1*	0.77	0.80	0.79
rs2439497	*CLU*	0.02	0.02	0.0010
rs2640734	*CLU*	0.05	0.05	0.0040
rs576256	*CLU*	0.12	0.04	0.0081
*rs11136000* *	*CLU*	0.33	0.66	0.78
rs2274567	*CR1*	0.76	0.12	0.18
rs9429940	*CR1*	0.15	0.89	0.20
rs17616	*CR1*	0.84	0.19	0.28
*rs6656401* *	*CR1*	0.75	0.39	0.52
rs638509	*PICALM*	0.0022	0.10	0.00098
rs694353	*PICALM*	0.00043	0.34	0.0010
rs10898433	*PICALM*	0.019	0.022	0.0012
*rs3851179* *	*PICALM*	0.74	0.61	0.54

Association with CSF ptau_181_ levels. P-values for association between the top three hits and CSF ptau_181_ levels in the Washington University (WU), Alzheimer's Disease Neuroimaging Initiative (ADNI) and Combined series.

*SNPs that are significant in previously reported genome-wide association studies are also shown, even when not ranked in the top three hits.

We failed to detect evidence for association between rs11136000 in *CLU*, which has been implicated in risk for AD, and CSF Aβ_42_ (p = 0.79) or ptau_181_ (p = 0.78) levels (Tables 3 and 4) in the combined sample. The top hits for CSF Aβ_42_ levels in *CLU* were rs10216623 (p = 0.011), rs2640734 (p = 0.036) and rs17057419 (p = 0.056); but these p-values do not pass multiple test correction. The top hits in *CLU* for association with CSF ptau_181_ were rs2439497 (p = 0.0010), rs2640734 (p = 0.004) and rs576256 (p = 0.0081) in the combined sample. The p-value threshold for Bonferroni correction for the entire study is 0.00021; therefore none of these p-values pass the multiple test correction. Set-based analyses of 57 SNPs from the *CLU* fine mapping set showed that there was evidence for association with ptau_181_ levels (p = 0.034). However this p-value is not significant after correction for the 4 SNP sets that were tested. There was no evidence for association in the set-based analyses for Aβ_42_ levels (p = 1). Haplotype analyses failed to identify significant association with either CSF phenotype.

The SNP in *CR1* that is implicated in risk for disease from recent GWAS is rs6656401. We failed to detect association between this SNP and either CSF Aβ_42_ (p = 0.63) or ptau_181_ (p = 0.52) levels in the combined sample. In *CR1* no SNPs were significant with either phenotype (table 3 and 4) and set-based analyses of the 24 SNPs within the *CR1* fine-mapping region provided no evidence for association (Aβ_42_ p = 1; ptau_181_ p = 1). Haplotype analyses failed to detect significant association with Aβ_42_ and ptau_181_.

Rs3851179, the *PICALM* SNP identified in the recent GWAS studies showed no evidence of association with either CSF Aβ_42_ or ptau_181_ levels (Tables 3 and 4). The top hits for CSF Aβ_42_ levels were rs7113656 (p = 0.0090), rs11234454 (p = 0.010) and rs10792828 (p = 0.011). The top hits for CSF ptau_181_ levels were rs638509 (p = 0.00098), rs694353 (p = 0.0010) and rs10898433 (p = 0.0012). Set-based analyses of 138 SNPs in the PICALM fine-mapping region failed to detect evidence for association with either Aβ_42_ (p = 0.56) or ptau_181_ (p = 0.47). Haplotype analyses also failed to identify significant association with these CSF phenotypes.

There is evidence of an interaction between SNPs in *PICALM* and *APOE ε4*, in at least one study the effects of risk associated SNPs in *PICALM* were found to be much stronger in the presence of the *APOE ε4* allele [6]. To investigate this interaction we included an interaction term for PICALM SNPs and the presence or absence of *APOE ε4* and performed association analyses between PICALM SNP genotypes and CSF Aβ_42_ and ptau_181_ in *APOE ε4* positive and *APOE ε4* negative substrata and using an *APOE ε4* by SNP interaction term in the combined sample. We failed to detect statistically significant associations in the *APOE ε4* negative and *APOE ε4* positive substrata and in the interaction analysis (table S2). The most significant p-value from these three analyses is for association of rs11234542 with CSF ptau_181_ levels in the *APOE ε4* negative substratum (p = 5.31×10^-5^; table S2). In this case the minor allele of rs11234542 was associated with higher CSF ptau_181_ levels.

Power to detect additive effects of more than an approximately 1.02 fold difference between the means was greater than 0.80 when alpha is 0.05 for all SNPs in this study. Even with an extremely conservative alpha of 0.00021 (Bonferroni correction for 233 tests) all SNPs in this study had power estimated at greater than 0.80 for at least a 1.05 fold difference (for reference significant association detected between rs2986019 in *CALHM1* on CSF Aβ_42_ levels by Kauwe et al was a 1.05 fold difference [19]).

## Discussion

While there were some suggestive associations of CSF ptau_181_ levels with *PICALM* SNPs, we failed to detect association that was significant after multiple test correction between SNPs in *BIN1, CLU, CR1* or *PICALM* and CSF Aβ_42_ or ptau_181_ levels in our analyses. The power calculations suggest that our single snp tests had a very high probability of detecting a strong, additive effect (1.05 fold difference) on CSF biomarker levels if it were present. The lack of significant associations suggests that there is not likely to be a strong additive genetic effect between the SNPs in this study and CSF levels of Aβ_42_ or ptau_181_. A recently published GWAS of 17 plasma lipoproteins in a sample of over 17,000 individuals identified 43 associated loci [24]. Close review of the results of that study shows that approximately one third of the significant associations show less than a 1.05-fold difference and about one sixth show less than a 1.03-fold difference. These findings suggest that small additive effects on protein levels are common and that much larger numbers of CSF samples will be required to precisely determine associations between Alzheimer's disease risk variants and biomarker levels. Greater sample sizes, while not immediately available, will be possible as we and other groups continue to collect additional specimens. Our set-based analyses suggest that there may be a signal for association with CSF ptau_181_ in the *CLU* gene region. This result, and the lack of signal with Aβ levels, are unexpected given data suggesting additive effects of CLU and APOE on Aβ deposition in mice [9]. The association is not significant after correction for the four sets that were tested but suggests that with increased power significant biomarker association may be detected.

An alternative interpretation of our results is that, given the lack of association with Aβ_42_ and ptau_181_, variants in these genes may modulate risk for AD through mechanisms that do not directly alter CSF levels of Aβ_42_ or ptau_181_. CLU, PICALM and CR1 participate in other processes not related to Aβ or tau aggregation, processing or clearance, and therefore studies of the role of these proteins in the brain may reveal evidence for additional disease mechanisms, which go beyond Aβ or tau accumulation. In fact there are several studies that link these genes with lipid metabolism and inflammatory pathways. Two of the identified AD susceptibility genes (*CLU, CR1*) have known functions in the immune system, which suggests a possible role for the immune system in the risk for AD. [25], [26]. Possible links between the genes in this study and lipid metabolism have also been identified and are reviewed by Jones et al [27].

Our study was designed specifically to detect additive genetic effects of common SNPs. Failure to detect significant association in this study design does not rule out, or even directly address, the possibility that these genes harbor rare variation that influence these biomarkers or that common variants in these genes have very small effects on these biomarkers. Finally, this approach may not detect complex, non-additive genetic mechanisms, such as complex gene-gene or gene-environment interactions that may modulate biomarker levels.

## Supporting Information

Table S1 **A complete list of SNPs in the study, position, minor allele frequencies (MAF), and p-values for association with CSF ptau_181_ and Aβ_42_ levels in the Washington University (WU), Alzheimer's Disease Neuroimaging Initiative (ADNI) and combined sample sets.** SNPs with values of #N/A failed to meet QC criteria.
(DOCX)

Table S2 **Association of SNPs in interaction with APOE ε4 alleles.** For each SNP in the PICALM gene region p-values for association with CSF ptau_181_ and Aβ_42_ levels for the SNP by presence/absence of the APOE ε4 allele interaction term, association in individuals without an APOE ε4 allele and association in individuals with an APOE ε4 allele are shown.
(DOCX)
