# Supplementary material for: Fine Mapping of Genetic Variants in BIN1, CLU, CR1 and PICALM for Association with Cerebrospinal Fluid Biomarkers for Alzheimer's Disease
Source: PLoS One. 2011 Feb 9;6(2):e15918. doi: 10.1371/journal.pone.0015918 (PMC3036586; doi:10.1371/journal.pone.0015918)
Supplement: Table S1 — A complete list of SNPs in the study, position, minor allele frequencies (MAF), and p-values for association with CSF ptau181 and Aβ42 levels in the Washington University (WU), Alzheimer's Disease Neuroimaging Initiative (ADNI) and combined sample sets. SNPs with values of #N/A failed to meet QC criteria. (DOCX) [file pone.0015918.s001.docx]

Supplemental table 1. A complete list of SNPs in the study, position, minor allele frequencies (MAF), and p-values for association with CSF ptau_181_ and Aβ_42_ levels in the Washington University (WU), Alzheimer’s Disease Neuroimaging Initiative (ADNI) and combined sample sets. SNPs with values of #N/A failed to meet QC criteria.

| **rs#** | **Chr.** | **Chromosome Position** | **MAF** | **Ptau_181_-WashU** | **Ptau_181_-ADNI** | **Ptau_181_ Combined** | **Aβ_42_-WashU** | **Aβ_42_-ADNI** | **Aβ_42_ Combined** |
| --- | --- | --- | --- | --- | --- | --- | --- | --- | --- |
| rs17615 | 1 | 205713085 | 0.3059 | 0.7665 | 0.2107 | 0.4969 | 0.7284 | 0.6315 | 0.5834 |
| rs4308977 | 1 | 205713521 | 0.3103 | 0.9267 | 0.2747 | 0.469 | 0.7858 | 0.4423 | 0.5435 |
| rs17616 | 1 | 205713546 | 0.3036 | 0.8426 | 0.1901 | 0.279 | 0.7771 | 0.5136 | 0.6024 |
| rs17258982 | 1 | 205719987 | 0.0701 | 0.4752 | 0.2927 | 0.9773 | 0.5864 | 0.6604 | 0.4306 |
| rs6540433 | 1 | 205720018 | 0.1492 | 0.7032 | 0.6911 | 0.5683 | 0.5008 | 0.4171 | 0.5105 |
| rs12021671 | 1 | 205724768 | 0.4009 | 0.7373 | 0.08675 | 0.5324 | 0.9468 | 0.02599 | 0.5634 |
| rs2182912 | 1 | 205726967 | 0.2006 | 0.6916 | 0.6461 | 0.8834 | 0.5921 | 0.3137 | 0.4645 |
| rs2182913 | 1 | 205727001 | 0.3567 | 0.633 | 0.7759 | 0.7544 | 0.6815 | 0.2605 | 0.5185 |
| rs9429940 | 1 | 205729535 | 0.1537 | 0.154 | 0.8917 | 0.199 | 0.7931 | 0.1597 | 0.5964 |
| rs1571344 | 1 | 205737551 | 0.1403 | 0.516 | 0.2889 | 0.3032 | 0.8688 | 0.5956 | 0.9051 |
| rs2025935 | 1 | 205742278 | 0.3128 | 0.1196 | 0.8127 | 0.4741 | 0.9667 | 0.5146 | 0.8272 |
| rs6656401 | 1 | 205758672 | 0.1879 | 0.7501 | 0.3871 | 0.5241 | 0.5505 | 0.7168 | 0.6284 |
| rs3886100 | 1 | 205805750 | NA | #N/A | #N/A | #N/A | #N/A | #N/A | #N/A |
| rs10127904 | 1 | 205814730 | 0.2669 | 0.9584 | 0.7169 | 0.6669 | 0.9042 | 0.848 | 0.9381 |
| rs2274567 | 1 | 205820244 | 0.1866 | 0.7646 | 0.1197 | 0.1754 | 0.5717 | 0.06541 | 0.7582 |
| rs3737002 | 1 | 205827396 | 0.2576 | 0.5255 | 0.7609 | 0.8372 | 0.6809 | 0.07218 | 0.9091 |
| rs12144461 | 1 | 205827427 | 0.01387 | #N/A | #N/A | #N/A | #N/A | #N/A | #N/A |
| rs17259045 | 1 | 205849330 | 0.097 | 0.005134 | 0.03995 | 0.5965 | 0.6973 | 0.07218 | 0.3846 |
| rs4844609 | 1 | 205849539 | 0.02077 | #N/A | #N/A | #N/A | #N/A | #N/A | #N/A |
| rs6691117 | 1 | 205849554 | 0.2719 | 0.4656 | 0.6629 | 0.9078 | 0.3894 | 0.1541 | 0.6873 |
| rs3818361 | 1 | 205851591 | 0.2138 | #N/A | #N/A | #N/A | #N/A | #N/A | #N/A |
| rs6701713 | 1 | 205852912 | 0.2198 | #N/A | #N/A | #N/A | #N/A | #N/A | #N/A |
| rs2296160 | 1 | 205861943 | 0.2026 | 0.9348 | 0.6456 | 0.5263 | 0.3219 | 0.7536 | 0.3327 |
| rs1408077 | 1 | 205870764 | 0.1949 | 0.4648 | 0.7337 | 0.9905 | 0.2776 | 0.6164 | 0.3686 |
| rs12080578 | 1 | 205887235 | 0.07308 | 0.4421 | 0.9472 | 0.6149 | 0.8114 | 0.8631 | 0.9191 |
| rs6704151 | 1 | 205888942 | 0.4635 | 0.8689 | 0.3086 | 0.5247 | 0.7667 | 0.08391 | 0.8881 |
| rs10863461 | 1 | 205907399 | NA | #N/A | #N/A | #N/A | #N/A | #N/A | #N/A |
| rs12567945 | 1 | 205908557 | 0.05252 | 0.7554 | 0.4454 | 0.4658 | 0.6081 | 0.6766 | 0.7762 |
| rs1048971 | 1 | 208712945 | 0.3649 | 0.2744 | 0.8306 | 0.5642 | 0.4748 | 0.7997 | 0.2517 |
| rs17258996 | 1 | 208720067 | 0.1952 | 0.9706 | 0.4098 | 0.4671 | 0.3807 | 0.9558 | 0.3157 |
| rs12476995 | 2 | 127512490 | 0.43 | #N/A | #N/A | #N/A | #N/A | #N/A | #N/A |
| rs7605172 | 2 | 127513584 | 0.1993 | 0.09545 | 0.6854 | 0.1601 | 0.3947 | 0.3193 | 0.9011 |
| rs3754617 | 2 | 127531305 | 0.1594 | #N/A | #N/A | #N/A | #N/A | #N/A | #N/A |
| rs2276582 | 2 | 127532894 | 0.4596 | 0.5227 | 0.6301 | 0.4366 | 0.2691 | 0.4281 | 0.4765 |
| rs2228955 | 2 | 127533165 | 0.02673 | #N/A | #N/A | #N/A | #N/A | #N/A | #N/A |
| rs1060743 | 2 | 127542003 | 0.301 | 0.4613 | 0.07471 | 0.09315 | 0.8282 | 0.495 | 0.8611 |
| rs3820757 | 2 | 127545411 | 0.4307 | 0.3676 | 0.9972 | 0.6258 | 0.1395 | 0.4253 | 0.3067 |
| rs17014873 | 2 | 127546689 | 0.07132 | 0.4757 | 0.2574 | 0.2382 | 0.6837 | 0.9165 | 0.7063 |
| rs753431 | 2 | 127546769 | 0.2452 | #N/A | #N/A | #N/A | #N/A | #N/A | #N/A |
| rs10194375 | 2 | 127556251 | 0.3699 | #N/A | #N/A | #N/A | #N/A | #N/A | #N/A |
| rs10200967 | 2 | 127558239 | 0.2518 | 0.2632 | 0.6011 | 0.205 | 0.4815 | 0.02117 | 0.5994 |
| rs17014923 | 2 | 127558400 | 0.1493 | #N/A | #N/A | #N/A | #N/A | #N/A | #N/A |
| rs4662706 | 2 | 127564971 | 0.01615 | #N/A | #N/A | #N/A | #N/A | #N/A | #N/A |
| rs11678252 | 2 | 127568167 | 0.437 | 0.3816 | 0.1277 | 0.1299 | 0.6024 | 0.005384 | 0.9471 |
| rs873270 | 2 | 127575888 | 0.1543 | 0.05424 | 0.8598 | 0.1259 | 0.8767 | 0.9162 | 0.7033 |
| rs749008 | 2 | 127575911 | 0.3606 | 0.259 | 0.3626 | 0.7138 | 0.4569 | 0.1276 | 0.9141 |
| rs9653202 | 2 | 127583577 | 0.06778 | 0.01892 | 0.8178 | 0.07683 | 0.8131 | 0.4229 | 0.8432 |
| rs10929009 | 2 | 127595309 | 0.2485 | 0.1098 | 0.9139 | 0.2986 | 0.7955 | 0.05068 | 0.978 |
| rs11904144 | 2 | 127596576 | 0.2438 | 0.4233 | 0.8473 | 0.4693 | 0.9137 | 0.09384 | 0.7732 |
| rs6431221 | 2 | 127601064 | 0.4948 | 0.059 | 0.7398 | 0.1013 | 0.6504 | 0.7821 | 0.8621 |
| rs744373 | 2 | 127611085 | 0.3097 | 0.7739 | 0.8033 | 0.792 | 0.4522 | 0.3241 | 0.4366 |
| rs17057419 | 8 | 27493305 | 0.178 | 0.6963 | 0.3566 | 0.792 | 0.08763 | 0.3846 | 0.05594 |
| rs6558008 | 8 | 27494223 | 0.2391 | 0.799 | 0.1848 | 0.2245 | 0.09377 | 0.6084 | 0.1349 |
| rs10216623 | 8 | 27496796 | 0.1317 | 0.7815 | 0.06322 | 0.1595 | 0.001073 | 0.8117 | 0.01099 |
| rs7828131 | 8 | 27496927 | 0.4137 | 0.4419 | 0.9658 | 0.4387 | 0.3317 | 0.5935 | 0.2058 |
| rs4732728 | 8 | 27497438 | 0.4412 | 0.6085 | 0.7467 | 0.455 | 0.6422 | 0.5539 | 0.5465 |
| rs7844965 | 8 | 27497981 | 0.224 | 0.5844 | 0.1143 | 0.4394 | 0.1504 | 0.7874 | 0.2158 |
| rs881146 | 8 | 27500194 | 0.0672 | 0.2986 | 0.5522 | 0.2795 | 0.2772 | 0.9521 | 0.3966 |
| rs10503813 | 8 | 27502817 | 0.2686 | 0.7557 | 0.3426 | 0.7564 | 0.2428 | 0.9384 | 0.1658 |
| rs7465418 | 8 | 27503241 | 0.0419 | #N/A | #N/A | #N/A | #N/A | #N/A | #N/A |
| rs6983452 | 8 | 27503945 | 0.4445 | 0.6038 | 0.7274 | 0.4478 | 0.868 | 0.8192 | 0.8442 |
| rs7012010 | 8 | 27504646 | 0.2946 | 0.8017 | 0.4796 | 0.8383 | 0.1261 | 0.7595 | 0.0979 |
| rs7012217 | 8 | 27504805 | 0.3294 | 0.3093 | 0.7237 | 0.5353 | 0.2265 | 0.5994 | 0.1199 |
| rs10111053 | 8 | 27506168 | 0.2598 | 0.7915 | 0.1698 | 0.5291 | 0.1173 | 0.3649 | 0.1528 |
| rs17466684 | 8 | 27508764 | 0.1788 | 0.1408 | 0.02976 | 0.842 | 0.4668 | 0.1715 | 0.4266 |
| rs2279591 | 8 | 27509680 | 0.3384 | #N/A | #N/A | #N/A | #N/A | #N/A | #N/A |
| rs10503814 | 8 | 27510492 | 0.05924 | #N/A | #N/A | #N/A | #N/A | #N/A | #N/A |
| rs9331949 | 8 | 27510603 | NA | #N/A | #N/A | #N/A | #N/A | #N/A | #N/A |
| rs9331942 | 8 | 27511031 | 0.03245 | #N/A | #N/A | #N/A | #N/A | #N/A | #N/A |
| rs3087554 | 8 | 27511359 | 0.1973 | #N/A | #N/A | #N/A | #N/A | #N/A | #N/A |
| rs2279590 | 8 | 27512170 | 0.3843 | 0.6695 | 0.2044 | 0.2365 | 1 | 0.08586 | 0.952 |
| rs9331930 | 8 | 27514211 | 0.2956 | 0.5632 | 0.8882 | 0.6976 | 0.182 | 0.9097 | 0.1988 |
| rs9331926 | 8 | 27517012 | 0.02774 | #N/A | #N/A | #N/A | #N/A | #N/A | #N/A |
| rs3216167 | 8 | 27517690 | 0.2853 | 0.6457 | 0.2656 | 0.6548 | 0.185 | 0.06861 | 0.2218 |
| rs9331908 | 8 | 27519535 | 0.3386 | 0.2439 | 0.2073 | 0.9763 | 0.1271 | 0.1111 | 0.1638 |
| rs11136000 | 8 | 27520436 | 0.3965 | 0.3298 | 0.6604 | 0.7761 | 0.9188 | 0.1439 | 0.7972 |
| rs4236673 | 8 | 27520846 | NA | #N/A | #N/A | #N/A | #N/A | #N/A | #N/A |
| rs1532278 | 8 | 27522232 | 0.375 | 0.9448 | 0.6002 | 0.6157 | 0.7424 | 0.379 | 0.6204 |
| rs867232 | 8 | 27523100 | 0.2688 | 0.9996 | 0.5339 | 0.7611 | 0.1382 | 0.9539 | 0.08691 |
| rs867231 | 8 | 27523309 | 0.2611 | 0.4705 | 0.7482 | 0.5522 | 0.1633 | 0.9693 | 0.09191 |
| rs9331896 | 8 | 27523603 | 0.402 | 0.3629 | 0.434 | 0.9947 | 0.9803 | 0.1587 | 0.8382 |
| rs2070926 | 8 | 27523738 | 0.3725 | 0.5873 | 0.2183 | 0.1835 | 0.9009 | 0.2278 | 0.8531 |
| rs867230 | 8 | 27524420 | 0.3767 | #N/A | #N/A | #N/A | #N/A | #N/A | #N/A |
| rs9331888 | 8 | 27524779 | 0.2894 | 0.6719 | 0.045 | 0.2305 | 0.2955 | 0.1181 | 0.3566 |
| rs10441615 | 8 | 27528666 | 0 | #N/A | #N/A | #N/A | #N/A | #N/A | #N/A |
| rs9314349 | 8 | 27530121 | 0.3942 | 0.5106 | 0.2282 | 0.1945 | 0.5052 | 0.2543 | 0.5954 |
| rs1982229 | 8 | 27530518 | 0.3833 | 0.9359 | 0.5098 | 0.6751 | 0.3813 | 0.1808 | 0.5844 |
| rs509778 | 8 | 27531056 | 0.009207 | #N/A | #N/A | #N/A | #N/A | #N/A | #N/A |
| rs7838352 | 8 | 27531812 | NA | #N/A | #N/A | #N/A | #N/A | #N/A | #N/A |
| rs569205 | 8 | 27532938 | 0.3811 | 0.3589 | 0.152 | 0.06584 | 0.6704 | 0.8736 | 0.7942 |
| rs484377 | 8 | 27533428 | 0.4244 | 0.1765 | 0.396 | 0.08588 | 0.7942 | 0.6371 | 0.7453 |
| rs2582367 | 8 | 27535944 | 0.4224 | 0.253 | 0.5447 | 0.1646 | 0.5599 | 0.5487 | 0.4565 |
| rs507341 | 8 | 27537903 | 0.2714 | 0.3921 | 0.2588 | 0.12 | 0.9809 | 0.5448 | 0.7373 |
| rs495150 | 8 | 27538273 | 0.2709 | 0.3893 | 0.284 | 0.134 | 0.3333 | 0.5839 | 0.5894 |
| rs499046 | 8 | 27538789 | 0.3419 | 0.1559 | 0.8698 | 0.2304 | 0.8249 | 0.6934 | 0.7962 |
| rs1493521 | 8 | 27539186 | 0.05983 | 0.5401 | 0.6541 | 0.4975 | 0.05106 | 0.4624 | 0.07992 |
| rs1493520 | 8 | 27539200 | 0.07069 | 0.3896 | 0.7181 | 0.3941 | 0.05352 | 0.3939 | 0.07692 |
| rs1389343 | 8 | 27540586 | 0.1429 | 0.706 | 0.3655 | 0.8087 | 0.9609 | 0.199 | 0.5904 |
| rs484458 | 8 | 27540871 | 0.2101 | 0.0961 | 0.2083 | 0.02691 | 0.4991 | 0.5758 | 0.3247 |
| rs525716 | 8 | 27541039 | 0.1359 | 0.5689 | 0.6709 | 0.3536 | 0.5145 | 0.4737 | 0.3826 |
| rs569214 | 8 | 27543709 | 0.3453 | 0.1164 | 0.5364 | 0.08642 | 0.9021 | 0.9309 | 0.8671 |
| rs520769 | 8 | 27545340 | 0.1243 | 0.1789 | 0.7922 | 0.1941 | 0.1022 | 0.1612 | 0.1239 |
| rs894019 | 8 | 27550531 | 0.3124 | #N/A | #N/A | #N/A | #N/A | #N/A | #N/A |
| rs17467992 | 8 | 27552600 | 0.4398 | 0.5543 | 0.6134 | 0.9576 | 0.6106 | 0.357 | 0.7912 |
| rs894021 | 8 | 27553511 | 0.02994 | #N/A | #N/A | #N/A | #N/A | #N/A | #N/A |
| rs577859 | 8 | 27553579 | 0.294 | #N/A | #N/A | #N/A | #N/A | #N/A | #N/A |
| rs500048 | 8 | 27553709 | 0.296 | 0.3313 | 0.04001 | 0.02999 | 0.5914 | 0.06453 | 0.4825 |
| rs12542107 | 8 | 27556346 | 0.4489 | #N/A | #N/A | #N/A | #N/A | #N/A | #N/A |
| rs10503815 | 8 | 27559479 | 0.07709 | 0.1963 | 0.8378 | 0.196 | 0.7993 | 0.2265 | 0.5524 |
| rs576256 | 8 | 27560008 | 0.2234 | 0.1171 | 0.03559 | 0.008062 | 0.09158 | 0.07448 | 0.08092 |
| rs17057494 | 8 | 27564204 | 0.11 | 0.9299 | 0.5753 | 0.6697 | 0.5145 | 0.1405 | 0.3477 |
| rs12677898 | 8 | 27568705 | 0.04708 | #N/A | #N/A | #N/A | #N/A | #N/A | #N/A |
| rs3779622 | 8 | 27569204 | 0.1079 | 0.7159 | 0.4681 | 0.824 | 0.3971 | 0.07598 | 0.1958 |
| rs552536 | 8 | 27569315 | 0.3277 | 0.3435 | 0.1627 | 0.08439 | 0.5407 | 0.4638 | 0.5415 |
| rs576956 | 8 | 27569528 | 0.3319 | 0.09235 | 0.09078 | 0.01484 | 0.4791 | 0.5608 | 0.5145 |
| rs542876 | 8 | 27576664 | NA | #N/A | #N/A | #N/A | #N/A | #N/A | #N/A |
| rs733078 | 8 | 27580941 | 0.1689 | 0.7295 | 0.4505 | 0.4909 | 0.7204 | 0.8993 | 0.4256 |
| rs4732600 | 8 | 27581224 | 0.01705 | #N/A | #N/A | #N/A | #N/A | #N/A | #N/A |
| rs17057523 | 8 | 27584365 | 0.08083 | 0.7718 | 0.8835 | 0.8668 | 0.2078 | 0.5042 | 0.1229 |
| rs485609 | 8 | 27586295 | 0.2878 | 0.3245 | 0.9523 | 0.402 | 0.8546 | 0.3637 | 0.6883 |
| rs1036710 | 8 | 27586394 | 0.07961 | 0.3032 | 0.3942 | 0.902 | 0.4965 | 0.6905 | 0.6503 |
| rs2640734 | 8 | 27588573 | 0.2266 | 0.04767 | 0.04563 | 0.004015 | 0.04528 | 0.07037 | 0.03596 |
| rs735277 | 8 | 27590646 | 0.4436 | #N/A | #N/A | #N/A | #N/A | #N/A | #N/A |
| rs559251 | 8 | 27595289 | 0.3462 | 0.3891 | 0.6606 | 0.6201 | 0.3528 | 0.1544 | 0.1469 |
| rs555300 | 8 | 27602770 | 0.2766 | 0.4806 | 0.7537 | 0.3832 | 0.6699 | 0.3452 | 0.4745 |
| rs7001584 | 8 | 27604084 | 0.4124 | #N/A | #N/A | #N/A | #N/A | #N/A | #N/A |
| rs17469137 | 8 | 27605162 | 0.04179 | #N/A | #N/A | #N/A | #N/A | #N/A | #N/A |
| rs3103908 | 8 | 27605340 | 0.4636 | 0.09277 | 0.04675 | 0.01059 | 0.3447 | 0.8841 | 0.6114 |
| rs2439497 | 8 | 27606641 | 0.225 | 0.01976 | 0.01736 | 0.001017 | 0.2245 | 0.3016 | 0.2797 |
| rs514196 | 8 | 27607836 | 0.4418 | 0.2034 | 0.1268 | 0.06051 | 0.1816 | 0.6438 | 0.2897 |
| rs7121363 | 11 | 85252758 | 0.1678 | 0.06642 | 0.8019 | 0.09875 | 0.2408 | 0.6171 | 0.06294 |
| rs36093844 | 11 | 85253775 | 0.2393 | 0.0642 | 0.9684 | 0.1322 | 0.1337 | 0.5415 | 0.02697 |
| rs11234454 | 11 | 85256952 | 0.4325 | 0.05955 | 0.05148 | 0.006233 | 0.008807 | 0.3378 | 0.00999 |
| rs7124956 | 11 | 85266467 | 0.2765 | 0.08282 | 0.6967 | 0.1043 | 0.0844 | 0.731 | 0.02198 |
| rs2084080 | 11 | 85268013 | 0.1453 | 0.03139 | 0.9557 | 0.08988 | 0.2346 | 0.762 | 0.05295 |
| rs7113656 | 11 | 85272387 | 0.2777 | 0.1169 | 0.6898 | 0.1332 | 0.05336 | 0.6883 | 0.008991 |
| rs12363602 | 11 | 85274049 | 0.2123 | 0.208 | 0.828 | 0.4278 | 0.4004 | 0.7837 | 0.1728 |
| rs17817355 | 11 | 85277593 | 0.1562 | #N/A | #N/A | #N/A | #N/A | #N/A | #N/A |
| rs12291381 | 11 | 85277758 | 0.245 | 0.4473 | 0.9812 | 0.5588 | 0.4615 | 0.7996 | 0.2458 |
| rs17744711 | 11 | 85278372 | 0.1582 | 0.1219 | 0.9619 | 0.2056 | 0.149 | 0.5957 | 0.02797 |
| rs12271536 | 11 | 85278483 | 0.2468 | 0.4383 | 0.9979 | 0.5685 | 0.2369 | 0.767 | 0.1299 |
| rs1445509 | 11 | 85280662 | 0.2706 | 0.394 | 0.9032 | 0.5674 | 0.04826 | 0.6729 | 0.04995 |
| rs1445508 | 11 | 85280768 | 0.2636 | 0.435 | 0.8925 | 0.6031 | 0.1242 | 0.888 | 0.1119 |
| rs10501600 | 11 | 85287207 | 0.4466 | 0.1462 | 0.05468 | 0.01521 | 0.08964 | 0.1823 | 0.1698 |
| rs7106460 | 11 | 85293197 | 0.2202 | 0.1287 | 0.8332 | 0.2079 | 0.02331 | 0.4305 | 0.01199 |
| rs17148585 | 11 | 85298471 | 0.317 | 0.3248 | 0.9809 | 0.3717 | 0.034 | 0.681 | 0.01199 |
| rs6592263 | 11 | 85299218 | 0.08032 | 0.4651 | 0.5083 | 0.9931 | 0.3156 | 0.3547 | 0.1698 |
| rs12285109 | 11 | 85299377 | 0.4356 | 0.09843 | 0.1813 | 0.02874 | 0.294 | 0.08031 | 0.3576 |
| rs1445496 | 11 | 85302626 | 0.423 | 0.1407 | 0.2076 | 0.04541 | 0.2951 | 0.1464 | 0.3437 |
| rs12274987 | 11 | 85304386 | 0.1484 | 0.9311 | 0.277 | 0.5733 | 0.648 | 0.3911 | 0.7253 |
| rs7927222 | 11 | 85304756 | 0.2393 | 0.4658 | 0.9621 | 0.7097 | 0.1696 | 0.9293 | 0.09091 |
| rs11603144 | 11 | 85312073 | 0.1816 | 0.709 | 0.01469 | 0.02419 | 0.667 | 0.008854 | 0.7902 |
| rs11825598 | 11 | 85312382 | 0.3694 | 0.858 | 0.1176 | 0.1566 | 0.7378 | 0.09195 | 0.8352 |
| rs12283410 | 11 | 85319603 | 0.1678 | 0.7013 | 0.2127 | 0.2601 | 0.5197 | 0.6904 | 0.6224 |
| rs7107303 | 11 | 85319833 | NA | #N/A | #N/A | #N/A | #N/A | #N/A | #N/A |
| rs7107881 | 11 | 85320411 | 0.4561 | 0.2661 | 0.03385 | 0.02082 | 0.06604 | 0.1154 | 0.04895 |
| rs597672 | 11 | 85330261 | 0.1698 | 0.4306 | 0.6854 | 0.9555 | 0.4543 | 0.3889 | 0.1339 |
| rs659018 | 11 | 85330336 | 0.3989 | 0.4442 | 0.53 | 0.2783 | 0.7666 | 0.2351 | 0.7163 |
| rs598561 | 11 | 85330474 | 0.4813 | 0.6023 | 0.9612 | 0.9319 | 0.3404 | 0.2178 | 0.8501 |
| rs661271 | 11 | 85330904 | 0.1724 | 0.9634 | 0.1284 | 0.2594 | 0.2006 | 0.3543 | 0.4006 |
| rs596864 | 11 | 85332235 | 0.4017 | 0.004164 | 0.1657 | 0.001774 | 0.08116 | 0.1784 | 0.09491 |
| rs7114678 | 11 | 85333822 | 0.1599 | #N/A | #N/A | #N/A | #N/A | #N/A | #N/A |
| rs11234491 | 11 | 85342151 | 0.1559 | 0.09304 | 0.9416 | 0.3619 | 0.97 | 0.586 | 0.6074 |
| rs1892943 | 11 | 85342820 | 0.217 | 0.01642 | 0.6693 | 0.06094 | 0.2664 | 0.8298 | 0.1788 |
| rs673751 | 11 | 85348593 | 0.3017 | 0.5044 | 0.842 | 0.6174 | 0.3863 | 0.2492 | 0.9031 |
| rs618679 | 11 | 85349350 | 0.1891 | 0.2263 | 0.3555 | 0.08808 | 0.6609 | 0.07289 | 0.7373 |
| rs2508690 | 11 | 85350170 | 0.441 | 0.02899 | 0.9288 | 0.1021 | 0.09525 | 0.1389 | 0.1499 |
| rs563773 | 11 | 85350918 | 0.4385 | 0.004851 | 0.9794 | 0.04563 | 0.2299 | 0.1013 | 0.3736 |
| rs17817582 | 11 | 85351248 | 0.2034 | 0.09038 | 0.8335 | 0.2949 | 0.3675 | 0.7456 | 0.1329 |
| rs10898427 | 11 | 85351694 | 0.1851 | 0.02453 | 0.5401 | 0.05574 | 0.09564 | 0.4701 | 0.08092 |
| rs11234495 | 11 | 85353082 | 0.2155 | 0.01039 | 0.8894 | 0.06653 | 0.2705 | 0.805 | 0.1688 |
| rs548121 | 11 | 85353181 | 0.1827 | 0.1662 | 0.2526 | 0.04913 | 0.3281 | 0.08737 | 0.7532 |
| rs510566 | 11 | 85355487 | 0.2468 | 0.1889 | 0.7817 | 0.397 | 0.993 | 0.2606 | 0.7842 |
| rs626542 | 11 | 85359768 | 0.1923 | 0.1161 | 0.319 | 0.0395 | 0.5939 | 0.2236 | 0.8162 |
| rs10501604 | 11 | 85361202 | 0.1516 | 0.1265 | 0.9045 | 0.4517 | 0.5421 | 0.9049 | 0.3097 |
| rs588380 | 11 | 85362583 | 0.4022 | 0.003635 | 0.3627 | 0.00431 | 0.1088 | 0.196 | 0.1099 |
| rs637304 | 11 | 85362814 | 0.1767 | 0.06471 | 0.4236 | 0.03765 | 0.4937 | 0.09267 | 0.96 |
| rs532470 | 11 | 85363744 | 0.4403 | 0.007784 | 0.702 | 0.03336 | 0.07098 | 0.15 | 0.1129 |
| rs475639 | 11 | 85367433 | 0.4662 | 0.002572 | 0.991 | 0.03164 | 0.3097 | 0.2642 | 0.3886 |
| rs7131120 | 11 | 85367660 | 0.1997 | 0.1257 | 0.6588 | 0.2125 | 0.6596 | 0.9678 | 0.5215 |
| rs615887 | 11 | 85367689 | 0.2334 | 0.08001 | 0.6388 | 0.3032 | 0.2748 | 0.0819 | 0.5654 |
| rs694353 | 11 | 85369829 | 0.4114 | 0.0004323 | 0.3435 | 0.001022 | 0.1274 | 0.1387 | 0.1069 |
| rs664050 | 11 | 85373254 | 0.4111 | 0.002049 | 0.3848 | 0.003469 | 0.2319 | 0.1056 | 0.2737 |
| rs585820 | 11 | 85374318 | 0.1844 | 0.156 | 0.2956 | 0.05693 | 0.8566 | 0.08972 | 0.5584 |
| rs669336 | 11 | 85376515 | 0.1994 | 0.1072 | 0.3734 | 0.5892 | 0.9968 | 0.2581 | 0.8212 |
| rs7938033 | 11 | 85379778 | 0.4187 | #N/A | #N/A | #N/A | #N/A | #N/A | #N/A |
| rs10792820 | 11 | 85381622 | 0.2296 | #N/A | #N/A | #N/A | #N/A | #N/A | #N/A |
| rs12795381 | 11 | 85381714 | 0.1159 | 0.7406 | 0.2134 | 0.2662 | 0.3923 | 0.6568 | 0.3956 |
| rs7951988 | 11 | 85391936 | 0.1884 | 0.1847 | 0.3676 | 0.07789 | 0.6268 | 0.08263 | 0.7552 |
| rs680119 | 11 | 85393680 | 0.3847 | 0.0158 | 0.2716 | 0.01113 | 0.1847 | 0.2319 | 0.2028 |
| rs10792821 | 11 | 85396289 | 0.1851 | 0.02381 | 0.738 | 0.09181 | 0.02237 | 0.7416 | 0.01698 |
| rs17148704 | 11 | 85398980 | 0.1428 | 0.2531 | 0.2628 | 0.077 | 0.4017 | 0.8786 | 0.2178 |
| rs4944552 | 11 | 85401454 | 0.2058 | 0.04789 | 0.7735 | 0.1106 | 0.2503 | 0.8531 | 0.07493 |
| rs648270 | 11 | 85404466 | 0.1816 | 0.1182 | 0.2268 | 0.03194 | 0.4584 | 0.09426 | 0.963 |
| rs519950 | 11 | 85404510 | 0 | #N/A | #N/A | #N/A | #N/A | #N/A | #N/A |
| rs642949 | 11 | 85407020 | 0.3606 | 0.005878 | 0.273 | 0.004742 | 0.128 | 0.8366 | 0.2188 |
| rs666682 | 11 | 85417486 | 0.4719 | 0.002514 | 0.843 | 0.02286 | 0.4536 | 0.1828 | 0.6364 |
| rs682058 | 11 | 85425002 | 0.2522 | 0.2025 | 0.9676 | 0.3421 | 0.7022 | 0.1263 | 0.4276 |
| rs10898431 | 11 | 85425229 | 0.2452 | 0.1232 | 0.4564 | 0.1428 | 0.1404 | 0.9196 | 0.02997 |
| rs664596 | 11 | 85429502 | 0.3491 | #N/A | #N/A | #N/A | #N/A | #N/A | #N/A |
| rs664629 | 11 | 85429531 | 0.4009 | 0.1086 | 0.1313 | 0.0166 | 0.1959 | 0.07789 | 0.3686 |
| rs17745273 | 11 | 85433186 | 0.2037 | 0.221 | 0.3673 | 0.1605 | 0.6269 | 0.679 | 0.2927 |
| rs1941375 | 11 | 85434781 | 0.2828 | 0.0565 | 0.3691 | 0.02812 | 0.5995 | 0.05467 | 0.8571 |
| rs677909 | 11 | 85435237 | 0.3083 | 0.346 | 0.9856 | 0.4473 | 0.9922 | 0.1245 | 0.3956 |
| rs4944555 | 11 | 85438264 | 0.1979 | 0.07436 | 0.7805 | 0.1616 | 0.3455 | 0.7252 | 0.09291 |
| rs669813 | 11 | 85439892 | 0.304 | 0.2171 | 0.9733 | 0.3308 | 0.6824 | 0.1056 | 0.2038 |
| rs9804630 | 11 | 85444230 | 0.187 | 0.09617 | 0.4017 | 0.05034 | 0.3861 | 0.1633 | 0.8112 |
| rs613222 | 11 | 85446753 | 0.4746 | 0.002383 | 0.8323 | 0.01658 | 0.5002 | 0.1195 | 0.7193 |
| rs11234532 | 11 | 85450284 | 0.09726 | 0.04672 | 0.1169 | 0.02136 | 0.503 | 0.1532 | 0.4535 |
| rs597446 | 11 | 85452707 | 0.4033 | 0.00468 | 0.1807 | 0.002486 | 0.1745 | 0.1459 | 0.1808 |
| rs1513390 | 11 | 85455860 | 0.4211 | 0.0008138 | 0.3054 | 0.001366 | 0.2032 | 0.202 | 0.2787 |
| rs2509608 | 11 | 85456945 | 0.1838 | 0.182 | 0.1759 | 0.03461 | 0.5212 | 0.06516 | 0.9301 |
| rs10898433 | 11 | 85458230 | 0.1282 | 0.01901 | 0.02165 | 0.001174 | 0.05441 | 0.06319 | 0.07393 |
| rs669556 | 11 | 85458970 | 0.1785 | 0.05214 | 0.4087 | 0.03796 | 0.1041 | 0.1736 | 0.3706 |
| rs621942 | 11 | 85461386 | 0.2519 | 0.1901 | 0.946 | 0.3634 | 0.2729 | 0.4093 | 0.2448 |
| rs638509 | 11 | 85462627 | 0.3857 | 0.002185 | 0.1046 | 0.0009821 | 0.5176 | 0.01344 | 0.9071 |
| rs536841 | 11 | 85465472 | 0.289 | 0.3652 | 0.9607 | 0.404 | 0.869 | 0.2127 | 0.5964 |
| rs7128598 | 11 | 85472779 | 0.2478 | 0.2394 | 0.8981 | 0.4802 | 0.1841 | 0.7655 | 0.1049 |
| rs565719 | 11 | 85473172 | 0.2853 | 0.4716 | 0.9388 | 0.4823 | 0.4198 | 0.1959 | 0.1449 |
| rs561655 | 11 | 85477927 | 0.312 | #N/A | #N/A | #N/A | #N/A | #N/A | #N/A |
| rs11234542 | 11 | 85480886 | 0.1546 | 0.0148 | 0.1163 | 0.004369 | 0.243 | 0.2108 | 0.2278 |
| rs572979 | 11 | 85480924 | 0.4835 | 0.03361 | 0.9586 | 0.127 | 0.4166 | 0.261 | 0.4735 |
| rs11825666 | 11 | 85482932 | 0.375 | #N/A | #N/A | #N/A | #N/A | #N/A | #N/A |
| rs645293 | 11 | 85487473 | 0.3237 | 0.9217 | 0.3493 | 0.554 | 0.5731 | 0.5531 | 0.2927 |
| rs12294130 | 11 | 85487527 | 0.2206 | 0.07653 | 0.8518 | 0.2094 | 0.4616 | 0.8113 | 0.2158 |
| rs11234545 | 11 | 85488111 | 0.06241 | 0.1254 | 0.1417 | 0.06361 | 0.2569 | 0.3497 | 0.1439 |
| rs17209931 | 11 | 85488474 | 0.06098 | 0.2103 | 0.0859 | 0.05889 | 0.3713 | 0.09514 | 0.2388 |
| rs629343 | 11 | 85488786 | 0.4236 | 0.06244 | 0.4087 | 0.5554 | 0.898 | 0.5729 | 0.9331 |
| rs542126 | 11 | 85488886 | 0.3493 | 0.8822 | 0.342 | 0.4578 | 0.1744 | 0.5269 | 0.06693 |
| rs495942 | 11 | 85490056 | 0.3287 | 0.341 | 0.1902 | 0.7729 | 0.8337 | 0.9311 | 0.6294 |
| rs17745409 | 11 | 85490433 | 0.1446 | 0.2495 | 0.1307 | 0.04572 | 0.01124 | 0.7699 | 0.04595 |
| rs11234546 | 11 | 85490603 | 0.09272 | 0.1025 | 0.05731 | 0.01187 | 0.6854 | 0.06016 | 0.5235 |
| rs1237999 | 11 | 85492678 | 0.3362 | 0.9772 | 0.4525 | 0.6464 | 0.1429 | 0.3239 | 0.05894 |
| rs11234548 | 11 | 85496398 | 0.2173 | 0.09025 | 0.8123 | 0.3595 | 0.3478 | 0.922 | 0.1429 |
| rs543293 | 11 | 85497725 | 0.3059 | 0.9006 | 0.5608 | 0.6511 | 0.2046 | 0.2042 | 0.07992 |
| rs34920413 | 11 | 85500972 | 0.4807 | #N/A | #N/A | #N/A | #N/A | #N/A | #N/A |
| rs646260 | 11 | 85501936 | 0.462 | 0.2286 | 0.8429 | 0.3828 | 0.2043 | 0.3428 | 0.05095 |
| rs659023 | 11 | 85502507 | 0.3551 | 0.8373 | 0.5161 | 0.8256 | 0.5032 | 0.4494 | 0.3277 |
| rs10792828 | 11 | 85504445 | 0.09233 | 0.09004 | 0.0255 | 0.004826 | 0.007446 | 0.02086 | 0.01099 |
| rs10792829 | 11 | 85505833 | 0.1222 | 0.02693 | 0.03509 | 0.002111 | 0.06624 | 0.02848 | 0.07792 |
| rs4944558 | 11 | 85507515 | 0.1871 | 0.7002 | 0.151 | 0.1541 | 0.4734 | 0.06113 | 0.6893 |
| rs567075 | 11 | 85507805 | 0.2942 | 0.5752 | 0.6528 | 0.8718 | 0.9793 | 0.2644 | 0.4745 |
| rs580887 | 11 | 85509081 | 0.4383 | 0.01763 | 0.5609 | 0.2293 | 0.7589 | 0.325 | 0.8591 |
| rs471470 | 11 | 85509189 | 0.3145 | 0.3854 | 0.6455 | 0.7095 | 0.487 | 0.165 | 0.2388 |
| rs472486 | 11 | 85509338 | 0.3263 | 0.4154 | 0.6297 | 0.7271 | 0.4341 | 0.2786 | 0.2547 |
| rs519961 | 11 | 85510072 | 0.3081 | 0.608 | 0.4234 | 0.8733 | 0.344 | 0.3462 | 0.1838 |
| rs17745474 | 11 | 85514617 | 0.2224 | 0.03641 | 0.8636 | 0.1323 | 0.3715 | 0.8716 | 0.1489 |
| rs7942719 | 11 | 85515017 | 0.147 | 0.1007 | 0.9396 | 0.1833 | 0.9726 | 0.9432 | 0.7123 |
| rs1898895 | 11 | 85515317 | NA | #N/A | #N/A | #N/A | #N/A | #N/A | #N/A |
| rs11234551 | 11 | 85515732 | 0.09958 | 0.1107 | 0.02392 | 0.007962 | 0.02054 | 0.01269 | 0.04096 |
| rs12292036 | 11 | 85516121 | 0.1271 | 0.154 | 0.727 | 0.4258 | 0.8913 | 0.9249 | 0.6583 |
| rs10792830 | 11 | 85516456 | 0.4644 | 0.02124 | 0.7887 | 0.06981 | 0.496 | 0.2905 | 0.6214 |
| rs7114401 | 11 | 85517583 | 0.377 | 0.3122 | 0.8345 | 0.4698 | 0.7946 | 0.2082 | 0.4256 |
| rs11607590 | 11 | 85517615 | 0.149 | 0.2108 | 0.9387 | 0.4922 | 0.313 | 0.4023 | 0.1149 |
| rs7926591 | 11 | 85518094 | 0.4447 | #N/A | #N/A | #N/A | #N/A | #N/A | #N/A |
| rs10898436 | 11 | 85518796 | 0.1913 | 0.7108 | 0.151 | 0.1647 | 0.5269 | 0.06113 | 0.7483 |
| rs10501610 | 11 | 85522134 | 0.1098 | 0.5684 | 0.4846 | 0.4591 | 0.1515 | 0.4885 | 0.07892 |
| rs4944560 | 11 | 85522693 | 0.4247 | 0.08765 | 0.5777 | 0.3806 | 0.9588 | 0.2116 | 0.6294 |
| rs12294949 | 11 | 85522870 | 0.05403 | 0.1073 | 0.7307 | 0.2389 | 0.3369 | 0.4385 | 0.6933 |
| rs7117280 | 11 | 85523477 | 0.2663 | 0.3585 | 0.5488 | 0.7658 | 0.4273 | 0.4437 | 0.3277 |
| rs3844143 | 11 | 85527891 | 0.473 | 0.04464 | 0.7713 | 0.2095 | 0.9644 | 0.1639 | 0.6384 |
| rs3894654 | 11 | 85529106 | 0.05903 | 0.2379 | 0.1414 | 0.7281 | 0.8861 | 0.08065 | 0.5624 |
| rs2374702 | 11 | 85529917 | 0.337 | 0.1656 | 0.0732 | 0.7674 | 0.7715 | 0.8866 | 0.5954 |
| rs2888903 | 11 | 85531824 | 0.4201 | 0.1421 | 0.5037 | 0.5119 | 0.8147 | 0.3691 | 0.8871 |
| rs7940019 | 11 | 85531942 | 0.1516 | 0.1034 | 0.8536 | 0.2405 | 0.1599 | 0.07789 | 0.1888 |
| rs7110631 | 11 | 85533835 | 0.2799 | 0.965 | 0.3759 | 0.5638 | 0.7771 | 0.3908 | 0.4416 |
| rs6592271 | 11 | 85534354 | 0.07966 | 0.3691 | 0.5324 | 0.9945 | 0.9962 | 0.9724 | 0.7922 |
| rs10898438 | 11 | 85535901 | 0.4506 | 0.2137 | 0.2086 | 0.932 | 0.9131 | 0.5307 | 0.6653 |
| rs12786057 | 11 | 85536065 | 0.34 | 0.2389 | 0.04996 | 0.5636 | 0.4316 | 0.5581 | 0.4486 |
| rs7941541 | 11 | 85536186 | 0.2904 | 0.9278 | 0.4559 | 0.7059 | 0.822 | 0.319 | 0.4016 |
| rs7480193 | 11 | 85539067 | 0.1878 | 0.5711 | 0.1767 | 0.1409 | 0.4878 | 0.0608 | 0.6244 |
| rs3862786 | 11 | 85540662 | 0.175 | 0.05284 | 0.8359 | 0.2423 | 0.7424 | 0.7668 | 0.4615 |
| rs56084917 | 11 | 85541928 | 0.03748 | #N/A | #N/A | #N/A | #N/A | #N/A | #N/A |
| rs6592272 | 11 | 85542839 | 0.2417 | 0.763 | 0.3465 | 0.5874 | 0.497 | 0.3186 | 0.5075 |
| rs10160337 | 11 | 85544219 | 0.1355 | 0.1008 | 0.884 | 0.1982 | 0.6929 | 0.6124 | 0.5584 |
| rs3851179 | 11 | 85546288 | 0.3367 | 0.7419 | 0.6143 | 0.5359 | 0.6392 | 0.354 | 0.963 |
| rs7113976 | 11 | 85547385 | 0.4411 | 0.293 | 0.8922 | 0.4206 | 0.9891 | 0.05031 | 0.6304 |
| rs7129687 | 11 | 85548832 | 0.458 | 0.5232 | 0.7783 | 0.8687 | 0.1095 | 0.8139 | 0.1099 |
| rs7107737 | 11 | 85550721 | 0.0608 | 0.1659 | 0.862 | 0.4932 | 0.1334 | 0.3833 | 0.1039 |
| rs7934191 | 11 | 85551897 | NA | #N/A | #N/A | #N/A | #N/A | #N/A | #N/A |
| rs11603136 | 11 | 85551970 | 0.4292 | 0.9279 | 0.5925 | 0.7006 | 0.1554 | 0.1612 | 0.1269 |
| rs11234568 | 11 | 85553870 | 0.4034 | 0.3382 | 0.2564 | 0.8448 | 0.5193 | 0.1677 | 0.6204 |
