# Supplementary material for: Fine Mapping of Genetic Variants in BIN1, CLU, CR1 and PICALM for Association with Cerebrospinal Fluid Biomarkers for Alzheimer's Disease
Source: PLoS One. 2011 Feb 9;6(2):e15918. doi: 10.1371/journal.pone.0015918 (PMC3036586; doi:10.1371/journal.pone.0015918)
Supplement: Table S2 — Association of SNPs in interaction with APOE ε4 alleles. For each SNP in the PICALM gene region p-values for association with CSF ptau181 and Aβ42 levels for the SNP by presence/absence of the APOE ε4 allele interaction term, association in individuals without an APOE ε4 allele and association in individuals with an APOE ε4 allele are shown. (DOCX) [file pone.0015918.s002.docx]

Supplemental table 2. Association of SNPs in interaction with APOE ε4 alleles. For each SNP in the PICALM gene region p-values for association with CSF ptau_181_ and Aβ_42_ levels for the SNP by presence/absence of the APOE ε4 allele interaction term, association in individuals without an APOE ε4 allele and association in individuals with an APOE ε4 allele are shown.

|  |  | **CSF ptau_181_** |  |  | **Aβ_42_** |  |
| --- | --- | --- | --- | --- | --- | --- |
| **SNP** | **APOE+/- by SNP Interaction** | **P-value in APOE4- stratum** | **P-value in APOE4+ stratum** | **APOE+/- by SNP Interaction** | **P-value in APOE4- stratum** | **P-value in APOE4+ stratum** |
| rs10160337 | 0.7614 | 0.3618 | 0.2662 | 0.6265 | 0.4951 | 0.9608 |
| rs10501600 | 0.6948 | 0.07858 | 0.0686 | 0.4964 | 0.1687 | 0.4712 |
| rs10501604 | 0.07977 | 0.07047 | 0.495 | 0.6031 | 0.5183 | 0.2054 |
| rs10501610 | 0.6498 | 0.6344 | 0.332 | 0.06831 | 0.01589 | 0.9631 |
| rs10792820 | 0.06241 | 0.003946 | 0.8081 | 0.9533 | 0.1881 | 0.225 |
| rs10792821 | 0.2967 | 0.03136 | 0.5241 | 0.7548 | 0.05785 | 0.1789 |
| rs10792828 | 0.1196 | 0.002504 | 0.5061 | 0.7098 | 0.04471 | 0.2367 |
| rs10792829 | 0.0714 | 0.0004341 | 0.4666 | 0.6604 | 0.1029 | 0.34 |
| rs10792830 | 0.1729 | 0.03678 | 0.9194 | 0.8051 | 0.5965 | 0.4822 |
| rs10898427 | 0.2499 | 0.02197 | 0.5879 | 0.884 | 0.1949 | 0.2284 |
| rs10898431 | 0.2023 | 0.6336 | 0.06972 | 0.9755 | 0.1742 | 0.08045 |
| rs10898433 | 0.07152 | 0.0002903 | 0.2917 | 0.6745 | 0.177 | 0.4917 |
| rs10898436 | 0.1182 | 0.05287 | 0.8258 | 0.8147 | 0.958 | 0.4751 |
| rs10898438 | 0.05408 | 0.3008 | 0.1257 | 0.9702 | 0.9466 | 0.9049 |
| rs11234454 | 0.5665 | 0.05242 | 0.02021 | 0.2321 | 0.01464 | 0.3365 |
| rs11234491 | 0.04752 | 0.03044 | 0.5344 | 0.3094 | 0.9184 | 0.2968 |
| rs11234495 | 0.0344 | 0.001356 | 0.9578 | 0.7321 | 0.3482 | 0.2922 |
| rs11234532 | 0.0107 | 0.0005794 | 0.855 | 0.6414 | 0.7725 | 0.3668 |
| rs11234542 | 0.01923 | 5.31E-05 | 0.5496 | 0.734 | 0.4269 | 0.3338 |
| rs11234545 | 0.04372 | 0.01036 | 0.6741 | 0.8186 | 0.1514 | 0.1972 |
| rs11234546 | 0.04027 | 0.0004598 | 0.9371 | 0.5885 | 0.2098 | 0.7019 |
| rs11234548 | 0.4566 | 0.75 | 0.3043 | 0.8619 | 0.2414 | 0.2983 |
| rs11234551 | 0.03237 | 0.0002231 | 0.6905 | 0.2225 | 0.01556 | 0.6387 |
| rs11234568 | 0.7613 | 0.801 | 0.9648 | 0.574 | 0.312 | 0.9457 |
| rs11603136 | 0.7883 | 0.8237 | 0.8923 | 0.7481 | 0.2941 | 0.858 |
| rs11603144 | 0.8948 | 0.1032 | 0.1525 | 0.1373 | 0.7316 | 0.1326 |
| rs11607590 | 0.9495 | 0.4851 | 0.8023 | 0.9609 | 0.1317 | 0.1942 |
| rs11825598 | 0.8631 | 0.4519 | 0.4238 | 0.4887 | 0.6857 | 0.5401 |
| rs12271536 | 0.1002 | 0.5038 | 0.1488 | 0.6622 | 0.7467 | 0.1455 |
| rs12274987 | 0.5722 | 0.9238 | 0.3546 | 0.3094 | 0.6757 | 0.3772 |
| rs12283410 | 0.5851 | 0.2169 | 0.5926 | 0.5662 | 0.2815 | 0.7488 |
| rs12285109 | 0.8696 | 0.07989 | 0.302 | 0.5669 | 0.2717 | 0.6018 |
| rs12291381 | 0.2097 | 0.7026 | 0.2434 | 0.7071 | 0.6063 | 0.09203 |
| rs12292036 | 0.4791 | 0.8399 | 0.2654 | 0.9762 | 0.7579 | 0.6616 |
| rs12294130 | 0.6149 | 0.399 | 0.2605 | 0.912 | 0.46 | 0.3085 |
| rs12294949 | 0.02779 | 0.04572 | 0.2684 | 0.9016 | 0.6108 | 0.7838 |
| rs12363602 | 0.08084 | 0.638 | 0.08458 | 0.308 | 0.8807 | 0.08574 |
| rs1237999 | 0.02588 | 0.5266 | 0.02834 | 0.5221 | 0.1697 | 0.3653 |
| rs12786057 | 0.4579 | 0.8496 | 0.2432 | 0.7519 | 0.5486 | 0.9479 |
| rs12795381 | 0.9142 | 0.3948 | 0.3857 | 0.2479 | 0.3879 | 0.4381 |
| rs1445496 | 0.7628 | 0.06466 | 0.3649 | 0.2769 | 0.13 | 0.9306 |
| rs1445508 | 0.467 | 0.9539 | 0.4496 | 0.7339 | 0.4298 | 0.3659 |
| rs1445509 | 0.9197 | 0.6174 | 0.782 | 0.6524 | 0.08858 | 0.1008 |
| rs1513390 | 0.2713 | 0.001314 | 0.2718 | 0.441 | 0.1353 | 0.955 |
| rs17148585 | 0.5483 | 0.5822 | 0.4267 | 0.5856 | 0.1024 | 0.3003 |
| rs17148704 | 0.7092 | 0.2727 | 0.1482 | 0.6056 | 0.4612 | 0.7501 |
| rs17209931 | 0.01123 | 0.001738 | 0.5199 | 0.7885 | 0.1274 | 0.4069 |
| rs17744711 | 0.2426 | 0.6917 | 0.1287 | 0.8432 | 0.1985 | 0.2381 |
| rs17745273 | 0.3978 | 0.4388 | 0.1264 | 0.6225 | 0.8631 | 0.2311 |
| rs17745409 | 0.5444 | 0.09401 | 0.03025 | 0.1805 | 0.03032 | 0.6543 |
| rs17745474 | 0.5895 | 0.3126 | 0.2023 | 0.6898 | 0.2214 | 0.3727 |
| rs17817355 | 0.3459 | 0.7568 | 0.1834 | 0.8028 | 0.1755 | 0.2109 |
| rs17817582 | 0.7068 | 0.3717 | 0.3575 | 0.6107 | 0.5327 | 0.1015 |
| rs1892943 | 0.09403 | 0.009649 | 0.8168 | 0.6317 | 0.505 | 0.3199 |
| rs1941375 | 0.9574 | 0.1395 | 0.2365 | 0.9819 | 0.9557 | 0.9856 |
| rs2084080 | 0.2362 | 0.3912 | 0.04503 | 0.5639 | 0.1305 | 0.2509 |
| rs2374702 | 0.5972 | 0.8959 | 0.4367 | 0.8337 | 0.8296 | 0.7772 |
| rs2508690 | 0.1312 | 0.04327 | 0.783 | 0.8851 | 0.2018 | 0.3694 |
| rs2509608 | 0.03393 | 0.009651 | 0.6603 | 0.2153 | 0.3364 | 0.1552 |
| rs2888903 | 0.1166 | 0.1907 | 0.4054 | 0.6217 | 0.5431 | 0.7798 |
| rs34920413 | 0.123 | 0.2788 | 0.2285 | 0.2582 | 0.5621 | 0.5674 |
| rs36093844 | 0.1678 | 0.569 | 0.0661 | 0.8572 | 0.2396 | 0.1016 |
| rs3844143 | 0.1908 | 0.1034 | 0.833 | 0.6616 | 0.6742 | 0.5903 |
| rs3851179* | 0.04414 | 0.522 | 0.05756 | 0.9336 | 0.7105 | 0.9642 |
| rs3862786 | 0.4675 | 0.1755 | 0.8795 | 0.8501 | 0.6115 | 0.3634 |
| rs3894654 | 0.9264 | 0.8607 | 0.9251 | 0.3755 | 0.9553 | 0.2952 |
| rs471470 | 0.1898 | 0.4629 | 0.3492 | 0.6749 | 0.3597 | 0.4979 |
| rs472486 | 0.01783 | 0.2049 | 0.06444 | 0.6942 | 0.451 | 0.5634 |
| rs475639 | 0.09247 | 0.006369 | 0.9524 | 0.8414 | 0.3505 | 0.3358 |
| rs4944552 | 0.496 | 0.2369 | 0.1287 | 0.633 | 0.3153 | 0.04979 |
| rs4944555 | 0.5197 | 0.316 | 0.1737 | 0.7377 | 0.5029 | 0.133 |
| rs4944558 | 0.07485 | 0.01859 | 0.8486 | 0.5992 | 0.4645 | 0.5892 |
| rs4944560 | 0.1391 | 0.1839 | 0.463 | 0.5916 | 0.8845 | 0.4191 |
| rs495942 | 0.5753 | 0.8568 | 0.4003 | 0.5488 | 0.6548 | 0.8296 |
| rs510566 | 0.6942 | 0.5739 | 0.8019 | 0.8253 | 0.967 | 0.8204 |
| rs519950 | NA | 1 | 1 | NA | 1 | 1 |
| rs519961 | 0.08605 | 0.6028 | 0.1106 | 0.7428 | 0.3861 | 0.4542 |
| rs532470 | 0.3221 | 0.02114 | 0.4741 | 0.809 | 0.1058 | 0.2022 |
| rs536841 | 0.01177 | 0.02219 | 0.2385 | 0.2439 | 0.6151 | 0.1046 |
| rs542126 | 0.03762 | 0.8107 | 0.02931 | 0.4375 | 0.1339 | 0.504 |
| rs543293 | 0.09713 | 0.7221 | 0.09544 | 0.8133 | 0.232 | 0.2498 |
| rs548121 | 0.07834 | 0.01269 | 0.9612 | 0.03904 | 0.1327 | 0.05669 |
| rs56084917 | 0.9873 | 0.2702 | 0.3404 | 0.7833 | 0.7764 | 0.535 |
| rs561655 | 0.06497 | 0.0927 | 0.5031 | 0.6794 | 0.777 | 0.9128 |
| rs563773 | 0.1669 | 0.01681 | 0.8429 | 0.7449 | 0.2483 | 0.5424 |
| rs565719 | 0.004069 | 0.03008 | 0.0879 | 0.7643 | 0.4666 | 0.5343 |
| rs567075 | 0.08982 | 0.3852 | 0.197 | 0.9696 | 0.7693 | 0.5229 |
| rs572979 | 0.05973 | 0.04385 | 0.3875 | 0.6722 | 0.7654 | 0.501 |
| rs580887 | 0.1198 | 0.09887 | 0.4538 | 0.6043 | 0.9917 | 0.6959 |
| rs585820 | 0.02981 | 0.00647 | 0.687 | 0.1472 | 0.595 | 0.05065 |
| rs588380 | 0.5199 | 0.004642 | 0.1744 | 0.4552 | 0.0796 | 0.787 |
| rs596864 | 0.2237 | 0.001005 | 0.297 | 0.7018 | 0.1286 | 0.5486 |
| rs597446 | 0.2983 | 0.002729 | 0.2486 | 0.4284 | 0.1543 | 0.9812 |
| rs597672 | 0.8239 | 0.8481 | 0.9014 | 0.9673 | 0.2097 | 0.5004 |
| rs598561 | 0.8139 | 0.9843 | 0.8008 | 0.1026 | 0.2084 | 0.3327 |
| rs613222 | 0.07864 | 0.002035 | 0.8653 | 0.8484 | 0.4984 | 0.5507 |
| rs615887 | 0.8994 | 0.574 | 0.6623 | 0.8061 | 0.3801 | 0.7114 |
| rs618679 | 0.1083 | 0.0235 | 0.941 | 0.1512 | 0.4215 | 0.07209 |
| rs621942 | 0.7816 | 0.8021 | 0.6404 | 0.8683 | 0.4388 | 0.7341 |
| rs626542 | 0.06893 | 0.007208 | 0.9841 | 0.1063 | 0.2907 | 0.07587 |
| rs629343 | 0.1353 | 0.2826 | 0.3099 | 0.4561 | 0.8358 | 0.4603 |
| rs637304 | 0.05337 | 0.007175 | 0.9477 | 0.07705 | 0.3157 | 0.05223 |
| rs638509 | 0.2884 | 0.00215 | 0.2608 | 0.7967 | 0.5616 | 0.804 |
| rs642949 | 0.3737 | 0.002352 | 0.1563 | 0.8858 | 0.2882 | 0.1787 |
| rs645293 | 0.05374 | 0.6135 | 0.07038 | 0.8831 | 0.6643 | 0.3547 |
| rs646260 | 0.7522 | 0.2857 | 0.2346 | 0.9839 | 0.1974 | 0.1524 |
| rs648270 | 0.07006 | 0.007266 | 0.9863 | 0.04482 | 0.1806 | 0.0394 |
| rs659018 | 0.8892 | 0.4714 | 0.6593 | 0.8096 | 0.9907 | 0.5146 |
| rs659023 | 0.07095 | 0.6114 | 0.09144 | 0.9779 | 0.797 | 0.4355 |
| rs6592263 | 0.5607 | 0.5826 | 0.5909 | 0.3165 | 0.1406 | 0.7206 |
| rs6592271 | 0.6477 | 0.8667 | 0.5999 | 0.5556 | 0.3576 | 0.9334 |
| rs6592272 | 0.4378 | 0.7463 | 0.1298 | 0.8863 | 0.7829 | 0.5447 |
| rs661271 | 0.892 | 0.2998 | 0.4031 | 0.01441 | 0.04284 | 0.1282 |
| rs664050 | 0.1763 | 0.001111 | 0.4432 | 0.8043 | 0.2822 | 0.7367 |
| rs664596 | 0.6044 | 0.2471 | 0.8653 | 0.3857 | 0.6833 | 0.381 |
| rs664629 | 0.5255 | 0.1731 | 0.07317 | 0.2955 | 0.2739 | 0.6798 |
| rs666682 | 0.1503 | 0.00718 | 0.7478 | 0.928 | 0.5668 | 0.6346 |
| rs669336 | 0.7499 | 0.9907 | 0.7793 | 0.9826 | 0.9136 | 0.9687 |
| rs669556 | 0.08891 | 0.008116 | 0.9487 | 0.08799 | 0.108 | 0.1567 |
| rs669813 | 0.02014 | 0.03648 | 0.2711 | 0.3483 | 0.8229 | 0.1427 |
| rs673751 | 0.03132 | 0.07637 | 0.2777 | 0.3492 | 0.3736 | 0.3411 |
| rs677909 | 0.02998 | 0.0631 | 0.2832 | 0.4471 | 0.6267 | 0.3077 |
| rs680119 | 0.388 | 0.005528 | 0.2475 | 0.3919 | 0.1103 | 0.8933 |
| rs682058 | 0.884 | 0.5881 | 0.9526 | 0.9675 | 0.6556 | 0.771 |
| rs694353 | 0.3462 | 0.002479 | 0.2247 | 0.5747 | 0.3512 | 0.1921 |
| rs7106460 | 0.6014 | 0.4486 | 0.246 | 0.4306 | 0.03285 | 0.1132 |
| rs7107737 | 0.09141 | 0.617 | 0.04775 | 0.4295 | 0.3007 | 0.9828 |
| rs7107881 | 0.7389 | 0.05119 | 0.06184 | 0.05292 | 0.01152 | 0.9505 |
| rs7110631 | 0.07282 | 0.6343 | 0.08321 | 0.4837 | 0.9805 | 0.2366 |
| rs7113656 | 0.1016 | 0.8424 | 0.04089 | 0.8997 | 0.1693 | 0.1202 |
| rs7113976 | 0.0608 | 0.1059 | 0.2671 | 0.3682 | 0.6454 | 0.276 |
| rs7114401 | 0.1246 | 0.1666 | 0.5377 | 0.9463 | 0.8041 | 0.6548 |
| rs7114678 | 0.05579 | 0.1581 | 0.2732 | 0.4703 | 0.4935 | 0.156 |
| rs7117280 | 0.3122 | 0.632 | 0.2954 | 0.8121 | 0.3036 | 0.5823 |
| rs7121363 | 0.2082 | 0.4398 | 0.05477 | 0.7381 | 0.3876 | 0.1172 |
| rs7124956 | 0.2048 | 0.6328 | 0.08024 | 0.57 | 0.3368 | 0.02355 |
| rs7128598 | 0.2938 | 0.9529 | 0.2675 | 0.7539 | 0.1893 | 0.1918 |
| rs7129687 | 0.1679 | 0.3785 | 0.3271 | 0.5648 | 0.1389 | 0.697 |
| rs7131120 | 0.02166 | 0.00796 | 0.4629 | 0.876 | 0.5317 | 0.4972 |
| rs7480193 | 0.1221 | 0.03857 | 0.9068 | 0.968 | 0.8406 | 0.794 |
| rs7926591 | 0.5796 | 0.9301 | 0.426 | 0.9008 | 0.7624 | 0.5381 |
| rs7927222 | 0.4529 | 0.9986 | 0.4902 | 0.4772 | 0.1554 | 0.3682 |
| rs7938033 | 0.1169 | 0.0001624 | 0.3156 | 0.5945 | 0.1475 | 0.1162 |
| rs7940019 | 0.9064 | 0.4663 | 0.7685 | 0.4785 | 0.8748 | 0.3384 |
| rs7941541 | 0.07396 | 0.6791 | 0.07198 | 0.9685 | 0.53 | 0.3669 |
| rs7942719 | 0.8718 | 0.2414 | 0.4232 | 0.6186 | 0.9579 | 0.4505 |
| rs7951988 | 0.01556 | 0.004628 | 0.5277 | 0.1746 | 0.3254 | 0.1171 |
| rs9804630 | 0.1984 | 0.01961 | 0.6415 | 0.03514 | 0.1089 | 0.03493 |
